# Relation between Anemia and Early Stages of Tuberculosis, with Reference to Treatment by Hypodermic Medication1Read before the Delta County Medical Society, December 14, 1906.

**Published:** 1907-04

**Authors:** B. R. Shurly

**Affiliations:** Detroit


### SELECTED ARTICLES.

### Relation Between Anemia and Early Stages of Tuberculosis, with Reference to Treatment by Hypodermic Medication.[1]

By B. R. SHURLY, M. D., Detroit.

[*Journal Michigan State Medical Society.*]

UNTIL a specific is obtained, the life of the tuberculous individual depends on early diagnosis and early treatment. A vast number of those infected with the pulmonary form pass through a so-called pretubercular or latent period. There is no tuberculosis without the tubercle bacilli, yet if we postpone our most strenuous efforts until the microscope reveals the lesions, the fight is beyond us. It is the cases of latent unrecognised tuberculosis with their peculiar interference of proper cell metabolism that demand more of our attention. There is frequently a prolonged prodromal period of impaired general condition that is marked by a well observed disturbance of the usual ratio between the body weight in pounds and the height in feet. A progressive loss in weight follows.

The external chest conditions, such as conformation, a limited expansion or capacity, may indicate the imperfect use of the pul-

---

1. Read before the Delta County Medical Society, December 14, 1906.

monary cells. The character of the pulse may give us additional evidence, such as acceleration with a relative decrease of arterial pressure. Lymphatism, with decidedly pathological tonsils, adenoids, and cervical glands, frequently exists. Will power replaces automatic power. Subjective symptoms become prominent—such as general malaise, failing digestion, weakness in the kness, and a slightly increased respiration. Where this prodromal period exists a varying degree of chloroanemia is frequently present. It is extremely marked in some cases, and the diagnosis is difficult in others.

The treatment of these anemias in associated or suspected tuberculosis is the direct subject of this paper. Medical literature provides us with many varieties of opinions upon the relations of chloroanemia and tuberculosis. It is agreed that so-called pretubercular conditions are generally attended by a diminution of hemoglobin and this is all out of proportion to the loss in red cells. Henocque characterises tubercular chloroanemias by a diminished supply of oxyhemoglobin. Incipient phthisis is usually marked by a slight leukocytosis, a slight reduction of red-blood corpuscles, and a moderate fall in hemoglobin. Twenty-nine per cent., according to Laache, of tubercular chloroanemias in the early stages show a diminished hemoglobin. Labbe regards chlorosis as a frequent first manifestation of a latent tuberculosis. Bramwell states that it is quite agreed that in families predisposed to tuberculosis, a stronger tendency to chlorosis exists. In his series of seventy-two cases of chlorosis, thirty per cent. showed definitely a tubercular family history.

It is extremely important that we should thoroughly examine and seriously treat all initial evidence of a slumbering tuberculosis. A number of patients suffering from various forms of anemias, with or without lymphatism, have come under my observation during the last two years, who have been treated with hypodermic medication of iron, arsenic, lecithin, sodium glycerophosphate, iodine, or guaiacol, in such combinations as the indications seemed to demand.

Rynd, in 1845, published in the Dublin Medical Press, the first report of medication administered under the skin. In 1850 Pravaz invented his well-known syringe that brought the hypodermic method into more general use. During the succeeding years, until 1880, only morphine and atropine seem to have had general recognition among the profession throughout the world. Roussel, of Paris, took up the work and added many new medicaments adapted to the subcutaneous method. The Bulletins of the French Society report the use of hypodermics of arsenic in 1883; antipyrin in 1884; salicylate of iron, arseniate of strychnine in 1886, and cyanide of mercury in 1890. During the next decade numerous articles appeared in the French and Italian literature, and the treatment has been generally accepted for more than fifteen years as exceedingly valuable, especially where iron or arsenic is indicated. I am unable to find a single article written in English with details upon this treatment. The therapeutic nihilist of the deepest dye admits without argument that iron, arsenic, and phosphorus have a wide field of usefulness.

When these valuable drugs are introduced hypodermically into the body through the lymphatic or capillary blood systems, the effects are more pronounced, more rapid, and quite as permanent as when administered by mouth. We know that the 38-45 grains of iron that are found in the human body are for the most part in complex chemical combination with the hemoglobin. This hemoglobin, as it was formerly claimed, required organic iron in its formation, and this idea prevails in the profession today, kindly and continuously nurtured by the enterprising firms who manufacture it in fancy forms. Whatever argument may be advanced in favor of organic iron by the stomach, I am confident that the ammoniated citrate or the arseniate of iron injected hypodermically in sufficient dosage will absolutely produce complete recovery from the pathologic blood conditions known as chlorosis, and that it will be found exceedingly efficient in many cases of secondary anemia.

It is estimated that the daily average of 100 milligrams of 1-6 grain of iron is required by the average individual, while many require only 6 milligrams per diem. It is generally conceded by physiological chemists that if inorganic iron is not rapidly assimilated it stimulates the iron producing function. It is therefore probable that the hypodermic method with the use of highly soluble forms of iron supplies directly both stimulation and iron to the deficient cells. Wherever iron is indicated, it may be given hypodermically in the form of the green ammoniated citrate or the arseniate of iron. Arsenic is best administered as Fowler's solution of the arseniate or the meta-arseniate of soda. These solutions may be combined, with or without the addition of glycerophosphates of sodium or calcium. Strychnine also may be used in combination with advantage. A valuable formula in chlorosis is citrate of iron, 0.05 grams; meta-arseniate of soda, 0.01 grams; glycerophosphate of soda, 0.2 grams; strychnine sulphate, 0.001 grams.

The French glass, 30 minim syringe of Luer, with its needle of good caliber, is very useful in this treatment. It is easily sterilised with hot water and quite durable, and it is not subject to the corrosive action of the metals. A needle kept in a glass jar or box for each individual removes any danger of infection. The injections are made into the deep cellular or muscular tissues of the buttocks or back. The stronger iron solutions are slightly painful. Chloroform or ether may be applied to the site of the puncture, if necessary. A blood examination should be made at the commencement and the completion of treatment. With a full dosage given daily an increase of ten per cent. in hemoglobin can be expected each week.

The green ammonio-citrate of iron has been used in the arts in this country to some extent, but not medicinally. It can be introduced into the system without danger in doses from 3-4 of a grain or .05 of a gram to 1.5 grains, or .1 gram daily. It is extremely soluble in water and can readily be taken up by the lymphatics and capillaries. The solution for hypodermic use must be sterilised and supplied in hermetically sealed bulbs, each containing one dose for immediate use. The splendid Italian product is preserved in cherry laurel water and imported in small white glass bulbs. Any cloudiness in the solution can be detected easily. After the patient is prepared, the slender neck of the bulb is broken, the sterile needle of our sterilised syringe is thrust into the bulb, which bulb is turned bottom side uppermost. The liquid can be then quickly drawn into the syringe.

A full dose of iron by the hypodermic method shows a reaction in from five to twenty minutes. The superficial capillaries dilate and a feeling of tension in the head is noted. Tingling sensations in the hands and feet or over the entire body may be detected. The pulse accelerates, the face is flushed, and a general feeling of well-being supervenes. In susceptible individuals or when a considerable over-dose is given, a wave of nausea follows, which may persist as continuous nausea for some hours. More than one and a half grains at a dose usually produces sudden vomiting within twenty minutes. From two and a half to five grains may produce several attacks of vomiting with great prostration for some hours. Treatment in the proper manner obviates all possibility of disturbing digestion. The teeth are preserved and no constipation results.

We recognise fresh air and forced feeding as our two sheet anchors in the treatment of tuberculosis, yet how few of our consumptives today reach the later stages of that disease without a course of creosote or its compounds in doses that have impaired the entire digestive apparatus? The conditions of the stomach will decide frequently the prognosis of our case, and tonic or palliative treatment that can be introduced hypodermically preserves the digestive tract for its proper functions. Drugs that impair digestion should be avoided.

The therapy of iron and arsenic is too well known to occupy the time of this society in argument as to the usefulness of these remedies. It suffices to say that the results of hypodermic medication will speak for themselves, if you will make the trial in well indicated cases. In the treatment of anemias associated with early tuberculosis, we can remember that we need not fear a localised tuberculosis as long as fresh air, good food and reconstructives or tonics maintain a general nutrition that will preserve the cell resistance of the pulmonary gland tissues.

CONCLUSIONS.

*First*—This method affords a rapid, safe and certain therapy, especially where iron, arsenic, phosphorus, or iodine is indicated. It does not interfere with other plans of treatment by mouth.

*Second*—Chlorosis will respond rapidly to this method alone.

*Third*—We can avoid gastrointestinal irritation, constipation, injury to the teeth, and imperfect assimilation.

*Fourth*—Drugs that impair digestion should be avoided in tubercular anemias.

*Fifth*—We are certain that the patient receives the medicine in the dosage we desire.

*Sixth*—The anemic patient is continuously under observation, as an average of thirty daily punctures is required, and a definite diagnosis of suspected tuberculosis or other conditions can then be made.

*Seventh*—Solutions of iron introduced into the subcutaneous cellular tissue in over-doses of one and a half grains will produce nausea or vomiting within twenty minutes.

*Eighth*—The hypodermic use of iron and arsenic is a well established and successful method of treatment that deserves recognition in this country.

DISCUSSION.

Dr. H. J. Hartz, Detroit, said the method as announced by Dr. Shurly is one requiring expert knowledge in its successful administration, and not easily carried out by the general practitioner. There are always certain dangers in the direct inoculation of drugs into the blood, and Dr. Hartz said that he would wish to avail himself of the screening action of the liver, in the administration of iron salts.

Dr. W. E. Coates, Manistee, confessed being a therapeutic nihilist. In regard to the efficacy of hypodermic medication he thought all workers in sanatoria were unanimous in the belief that medicinal methods were of very little use except in emergencies. Over-dosing is a frequent cause of impaired digestion. Dr. Coates questioned the use of the term chlorosis in this form of anemia. Chlorosis is probably a specific disease, although it may be associated with tuberculosis in its early stage. The usual anemia is a secondary change. He believes in fresh air treatment in all cases and thinks that just as much could have been accomplished for Dr. Shurly's patients by these means.

Dr. E. L. Shurly, Detroit, said that it is the great tendency of nature to heal disease, so that the results regarding any one method of treatment are apt to be fallacious. He had personally found the hypodermic method of medication a very valuable help in treating tuberculosis. He had given a varied list of drugs in this way.

Dr. George Dock, Ann Arbor, said that the paper was interesting because it called attention to an exact method of treatment. He objected to the reintroduction of the discarded term "pretubercular," which means simply presputum stage.

The tuberculous subject is very often anemic, this anemia frequently appearing before evidence of physical signs and sputum. There is a peculiar pallor about many of these patients. The hemoglobin may test 80 per cent. when the patient looks quite as pale as a pernicious anemia patient with 30 per cent. to 40 per cent. hemoglobin. One must first make certain of the condition of the blood before drawing conclusions.

The anemia is usually secondary. It is very erroneous to consider the condition chlorosis. The tuberculous patient may look pale and not need treatment for that condition. The cases with most marked anemia may not need iron. Fresh air and diet is usually sufficient. There is no doubt but that in certain cases iron is very useful. Taken internally it can be absorbed if given in the proper form. The advantage of the hypodermic method is its accuracy and the chance for observation of the patient. There is no reason why iron should not be given in this way except that it is not necessary. Iron and arsenic can both be given satisfactorily by the mouth.

Dr. B. R. Shurly, Detroit, in closing said that the danger of infection was no greater than in giving morphine hypodermically. The advantages were the more definite dosage and prevention of irritation to the stomach. Many preparations of iron are inert, and it is always a question of how much is absorbed, unless the preparation is quite fresh. Dr. Shurly has had a number of cases in his practice who could not take iron by mouth without upsetting the stomach. In these cases, where there is chlorosis, the hypodermic method of administering iron is very valuable, such cases improving rapidly under the treatment. Dr. Shurly would not do away with other methods of treatment. Hypodermic medication does not interfere with them.

